# Surveillance of influenza A and B viruses from community and hospital wastewater treatment plants

**DOI:** 10.1111/1758-2229.13317

**Published:** 2024-08-22

**Authors:** Sneka Panneerselvam, Athira Manayan Parambil, Anup Jayaram, Prasad Varamballi, Chiranjay Mukhopadhyay, Anitha Jagadesh

**Affiliations:** ^1^ Manipal Institute of Virology Manipal Academy of Higher Education Manipal India

## Abstract

Influenza virus is a well‐known pathogen that can cause epidemics and pandemics. Several surveillance methods are being followed to monitor the transmission patterns and spread of influenza in the community. Wastewater‐based Epidemiology (WBE) can serve as an additional tool to detect the presence of influenza viruses. The current study primarily focuses on surveillance of Influenza A and Influenza B in wastewater treatment plant (WWTP) samples. A total of 100 wastewater samples were collected in July (*n* = 50) and August (*n* = 50) 2023 from four different WWTPs in Manipal and Udupi, district of Karnataka, India. The WWTP samples were processed and tested by Real‐Time reverse transcriptase PCR (RT‐PCR). The data generated was analysed in comparison with the clinical Influenza cases. Of the 100 samples, 18 (18%) tested positive for Influenza A virus and 2 (2%) tested positive for Influenza B virus, with a viral load ranging 1.4 x 10^2^–2.2 x 10^3^ gc/L for influenza A virus and 5.2 x 10^3^–7.7 x 10^3^gc/L for influenza B virus. On correlating the WWTP positivity with clinical case, it was found that influenza clinical cases and virus positivity in wastewater increased simultaneously, emphasizing WBE as a concurrent method for monitoring influenza virus activity.

## Graphical abstract

In this study, wastewater‐based epidemiology (WBE) was used as a tool for surveillance of influenza A and B viruses. Samples collected from WWTPs were subjected to molecular testing for the presence of Influenza A and Influenza B viruses. The resultant data were then correlated with clinical cases from the catchment area to identify the presence and trends of influenza A and B viruses within the population.

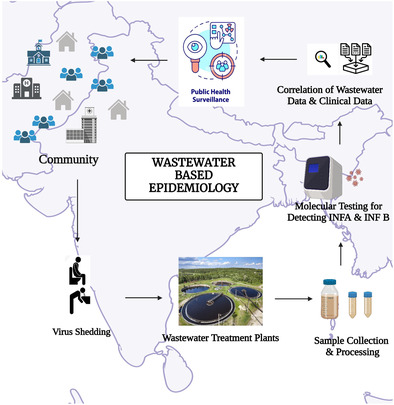

## INTRODUCTION

The influenza virus belongs to the family *Orthomyxoviridae* and contains negative‐sense, single‐stranded segmented RNA. Influenza is highly contagious and mainly spreads through respiratory secretions (Influenza|CDC Yellow Book 2024, n.d.). Every year, large numbers of people in the population are infected by influenza viruses (Knipe & Peter, 2013). Surveillance remains a fundamental tool for monitoring the circulating virus. In public health, surveillance is a systematic approach for collecting data, in which the data is analysed and interpreted. The interpreted data gives information for monitoring, planning, and evaluating the interventions and public health policies (Surveillance in emergencies, n.d.; Buckeridge & Cadieux, 2006). Influenza surveillance primarily helps to identify the influenza virus prevalence and circulating strains that further helps to manage the transmission and also determine the components of vaccines for mitigating interventions (Brammer et al., 2009; Influenza (seasonal), n.d.; Hannoun, 1993). Globally, influenza surveillance is carried out by the Global Influenza Surveillance and Response System (GISRS) for monitoring transmission patterns to develop preparedness plans and action against zoonotic, seasonal, and pandemic influenza viruses. Furthermore, GISRS serves as a global warning system for identifying the circulating strains of Influenza (Influenza (seasonal), n.d.; Global Influenza Surveillance and Response System (GISRS), n.d.).

In India, Integrated Disease Surveillance Program (IDSP) under the National Centre for Disease Control (NCDC) monitors prepares intervention and responses against influenza in humans (Integrated Disease Surveillance Programme (IDSP), n.d.). Wastewater‐based epidemiology (WBE) can act as a complementary approach to the current surveillance of the influenza virus in the community as the whole population in a community contributes to the wastewater collected in wastewater treatment plants (WWTPs) (Sims & Kasprzyk‐Hordern, 2020). Poliovirus was first detected in wastewater and later, other enteric viruses such as hepatitis A, hepatitis E, and rotavirus were also detected (Farkas et al., 2018; Gholipour et al., 2022; Metcalf & Melnick, 1995). Initial research has been focused mainly on the detection of enteroviruses in WWTPs. However, later studies have concluded the presence of various respiratory viruses such as adenovirus, influenza virus, coronavirus etc. in wastewater (Foladori et al., 2020; Kitajima et al., 2020; Lowry et al., 2023; Toribio‐Avedillo et al., 2023). In India, research on detecting poliovirus in wastewater has been ongoing for more than two decades (Deshpande et al., 2003; Tiwari & Dhole, 2018). Wastewater‐based study for respiratory viruses such as SARS‐CoV‐2 has also been initiated across India (Dharmadhikari et al., 2022; Joshi et al., 2022). However, using wastewater as a surveillance tool for screening influenza virus has not been studied in India. Thus, the primary objective of the study was wastewater‐based surveillance of Influenza A and Influenza B viruses. This involved collecting and analysing samples from WWTPs in both community and hospital WWTPs. The aim was to establish a reliable method that can help in early detection, track prevalence, and potentially predict outbreaks of these viruses within the community.

## EXPERIMENTAL PROCEDURES

### Sampling criteria and sample collection

The inlet wastewater samples were collected from four different WWTPs in Udupi district. The WWTP were selected such that population coverage includes only hospital, only community, and mixed (community and hospital). WWTP 1 (End Point New, Manipal) and WWTP 2 (End Point Old, Manipal) each have a daily capacity of 1800 cubic metres with population coverage around 15,000 residents and a tertiary hospital with approximately 1000 beds. WWTP 3 (Manipal Institute of Technology (MIT) Campus, Manipal) has a daily capacity of 2000 cubic metres with approximate population coverage of 15,000 residents. WWTP 4 (TMA Pai Hospital, Udupi) has a daily capacity of 50 m^3^ for a 200 beds hospital. The wastewater samples were collected on alternate days between 8 and 11 am and transported to the laboratory in cold chain at 2–8°C. A total of 100 wastewater samples collected from the four WWTPs during July and August 2023 (50 from each month) were included in the study.

### Sample processing

**FIGURE 1:**
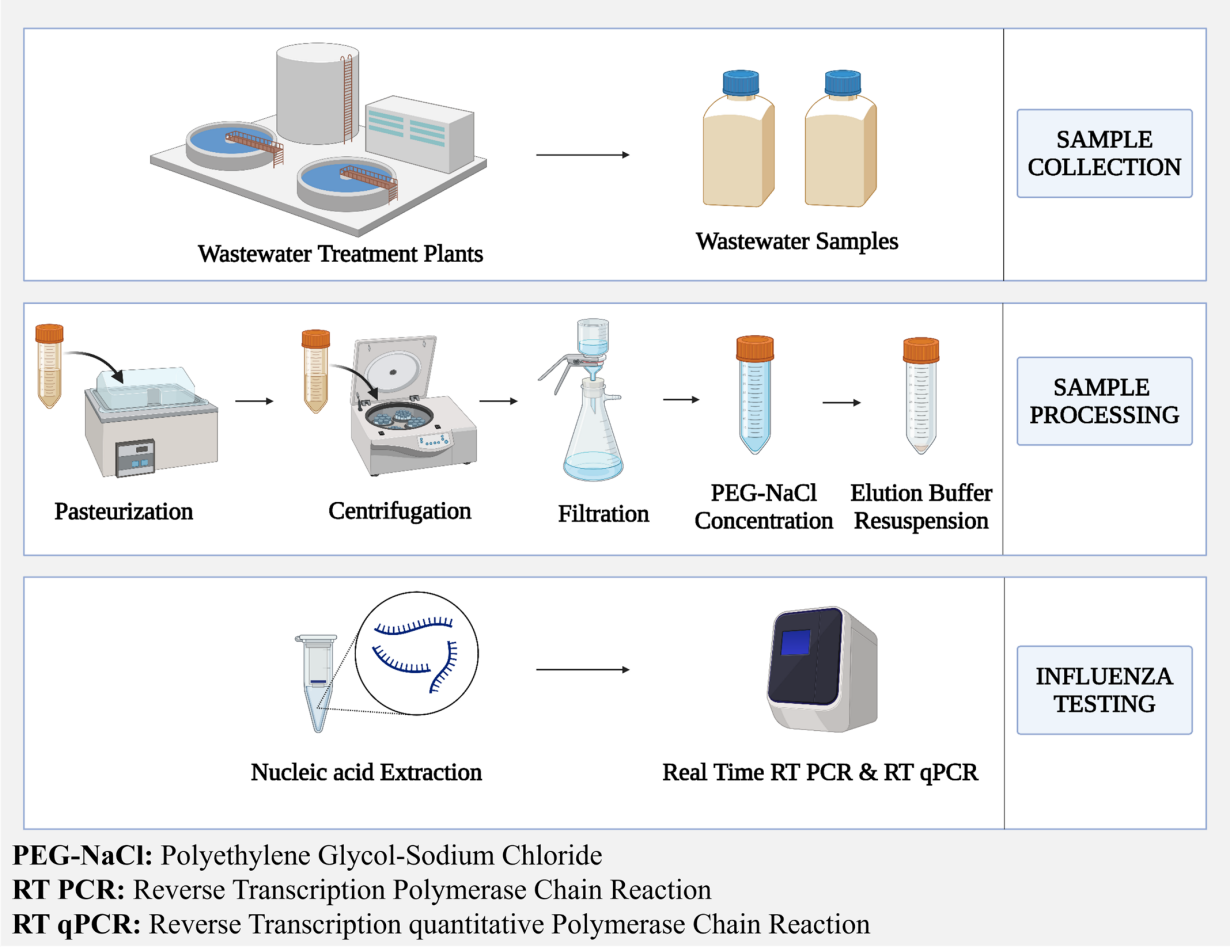
Illustration of workflow for processing of samples collected from wastewater treatment plant (WWTPs).

The wastewater samples were pasteurized for 1 h at 60°C in a water bath to ensure worker safety. Following pasteurization 45 mL of sample was transferred to a fresh tube and centrifuged at 4°C for 10 min at 4500 rpm in the cold centrifuge to separate solids and remove larger debris. Immediately after centrifugation, the supernatant was filtered using a vacuum unit assembly with Whatman filter paper grade no. 1 to remove large organic matter. Filtrate was then passed through polyethersulphone (PES) membrane filter, with a pore size of 0.8 μm followed by another membrane of size 0.22 μm for removing the smaller impurities. The filtered 40 mL wastewater samples were concentrated using the PEG‐NaCl precipitation method, 8% (3.2 g) of PEG 8000 and 1.7% (0.68 g) of NaCl were added to the samples to concentrate the viral particles by causing them to precipitate out of solution. The samples with PEG‐NaCl were kept in a shaker water bath at 4°C for 1–2 h for thorough mixing and precipitation. After concentration, samples were centrifuged at 4500 rpm for 1 h at 4°C. The supernatant of the samples was discarded without disturbing the pellet to remove liquids and to preserve the concentrated particles. The pellet was resuspended with 140 μL of elution buffer and allowed to dissolve for 5 min for dissolving and stabilizing the concentrated particles. The suspension was mixed thoroughly and used for nucleic acid extraction. The brief workflow of sample collection and processing are illustrated in Figure 1.

### Real‐time reverse transcriptase PCR (RT‐PCR) for detection of influenza RNA

Nucleic acid from WWTP samples was extracted using the QIAGEN Viral RNA kit (Qiagen, Germany) with final elution volume of 40 μL. The extracted nucleic was tested using a Hi‐PCR® COVID FLU RSV Multiplex Probe PCR kit (HiMedia, India). The Multiplex Probe PCR kit contains two primer‐probe mixes. One primer‐probe mix targets influenza A, influenza A(H1N1)pdm09, SARS‐CoV‐2, and Internal control (IC), and the other mix targets influenza A(H3N2), respiratory syncytial virus (RSV), influenza B virus and IC. Real‐Time Reverse Transcriptase PCR (RT‐PCR) was performed according to the manufacturer's instructions using QuantStudio™ 5 Real‐Time PCR system (Applied Biosystems, USA).

### Preparation of standards for quantification of influenza viral load

Nucleic acid extracted from Influenza A virus/Guangdong‐Maonan/SWL1536/2019 and Influenza B/Phuket/3073/2013 standard strains procured from International Reagent Resource (IRR) were used for preparation of standards. Conventional PCR was carried using CDC‐recommended primers (Table 1). The assay components included master mix (12.5 μL) (2×), forward primer (0.5 μL) (10 pmol/μL), reverse primer (0.5 μL) (10 pmol/μL), enzyme (1 μL) (25×), and template (5 μL), making a total volume of 15 μL. The standardized cycling condition for PCR was 50°C for 30 min (1 cycle), 95°C for 10 min (1 cycle), followed by 45 cycles of amplification (95°C for 15 s, 55°C for 30 s, 72°C for 30 s), and final extension of 72°C for 2 min (1 cycle), and 4°C for ∞.

**TABLE 1:** Primers and probes used for real‐time reverse transcriptase polymerase chain reaction (RT‐PCR).

List of primers and probes		
S. No	Type	Sequences
1.	Influenza A‐F	GACCRATCCTTCACCTTGAC
2.	Influenza A‐R	AGGGCATTYTGGACAAAKCGICTA
3.	Influenza A‐Probe	(FAM)TGCAGTCCTCGCTCACTGGGCACG(BHQ)
4.	Influenza B‐F	TCCTCAAYTCACTCTTGAGCG
5.	Influenza B‐R	CGGTGCTCTTGACCAAATTGG
6.	Influenza B‐Probe	(FAM)CCAATTCGAGCAGCTGAAACTGCGGTG(BHQ)

Gel for electrophoresis was prepared using 0.8% of agarose in TBE buffer. PCR products and DNA ladder were loaded and electrophoresis was carried out at 120 V for 90 min. The desired product size of Influenza A gene was 106 bp, whereas that of Influenza B gene was 103 bp. The gel bands were extracted and purified using the GenElute™ (Sigma‐Aldrich, USA) gel extraction kit. The purified products were quantified using the Nanodrop® 2000 spectrophotometer and Qubit fluorometer (Qubit dsDNA HS Assay kit, Invitrogen, USA). The concentration of Influenza A was 27.2 (ng/μL) and Influenza B was 2.1 (ng/μL). The copy number determined for Influenza A and Influenza B was 2.341 x 10^11^ copies/μL and 1.86 x 10^10^ copies/μL, respectively. The purified standards were aliquoted for one time use and stored at −20°C and used within one week.

### Standardization of real‐time quantitative PCR

Prepared standards were diluted in logarithmic dilutions and 10^−9^, 10^−10^, 10^−11^ and 10^−12^ dilutions were used as standards for influenza A, whereas dilutions 10^−7^, 10^−8^, 10^−9^, and 10^−10^ were used for generating standard curve for influenza B virus genes in triplicates using CDC‐recommended primers and probes (Table 1). The assay components included master mix (12.5 μL) (2×) and enzyme (1 μL) (25×) from Applied Biosystems, and primers (0.5 μL) (10 pmol/μL) and probes (0.5 μL) (2 pmol/μL). Cycling conditions included the reverse transcription process at 50°C for 30 min and initial denaturation at 95°C for 15 s, followed by amplification for 45 cycles (denaturation at 95°C for 30 s and annealing and extension at 55°C for 30 s).

Amplification plots and standard curves of triplicates for selected dilutions of Influenza A and B standards were analysed and the run was validated. Validity parameters for Influenza A and B were as follows: slope: −3.00 to −3.70; *R* ^2^ >0.98; efficiency: 85–115% (Table 2). The Limit of detection (LoD) i.e. the lowest copy number detected for Influenza A and B virus genes was of 0.23 copies/μL and 1.86 x 10^1^ copies/μL of reaction respectively.

**TABLE 2:** Standard curve and validity parameters for Influenza A and B virus RNA.

Influenza A virus RNA		
Dilutions	Average *C*_t_ value	Copies/μL
10^−9^	32.53	2.341 x 10^2^
10^−10^	35.50	2.341 x 10^1^
10^−11^	37.63	2.341
10^−12^	39.92	0.2341

Influenza B virus RNA		
Dilutions	Average *C*_t_ value	Copies/μL
10^−6^	26.46	1.86 x 10^4^
10^−7^	30.14	1.86 x 10^3^
10^−8^	33.57	1.86 x 10^2^
10^−9^	36.51	1.86 x 10^1^

Standard curve					
Target	Slope	Y‐interpreted	*R* ^2^	Efficient (%)	Error
Inf A	−3.011	39.089	0.995	114.824	0.147
Inf B	−3.321	39.594	0.993	100.051	0.195

WWTP samples that tested positive for Influenza A and B using the Multiplex Probe PCR kit were additionally tested by the standardized quantitative PCR using QuantStudio™ 5 Real‐Time PCR system (Thermo Fischer Scientific, USA) and viral load was estimated as gene copies/L by calculating the sample input and elution volume (Amereh et al., 2022).

### Clinical cases

Manipal Institute of Virology (MIV) serves as testing centre for the diagnoses of various viral etiologies. Suspected influenza cases were sent to the laboratory for testing, with throat swab collected from patients. These samples were processed and tested using RealStar® Influenza Screen & Type RT‐PCR Kit (altona Diagnostics, Germany) for detecting influenza (H1N1)pdm09, influenza A(H3N2) and Influenza B. The month‐wise positive cases (July and August) of Influenza A virus and Influenza B virus from Udupi district as well as positivity among WWTP catchment area were used for correlation analysis.

## RESULTS

### Prevalence of influenza A and B virus in WWTP samples

Out of the 100 WWTP samples tested with Hi‐PCR® COVID FLU RSV Multiplex Probe PCR kit, 18 (18%) samples tested positive for influenza A virus and two (2%) samples tested positive for influenza B virus. Among July 2023 samples, only 1 (2%) out of 50 WWTP samples tested positive for influenza A virus, whereas 17 of 50 August 2023 samples tested positive, resulting in a 34% positivity rate. All the 18 influenza A positive WWTP samples tested positive for influenza A(HIN1)pdm09, however, four samples tested positive for both influenza A(HIN1)pdm09 and influenza A(H3N2) virus. Influenza B virus positivity in July was 0%, however, in August, two (4%) of the 50 samples tested positive.

**FIGURE 2:**
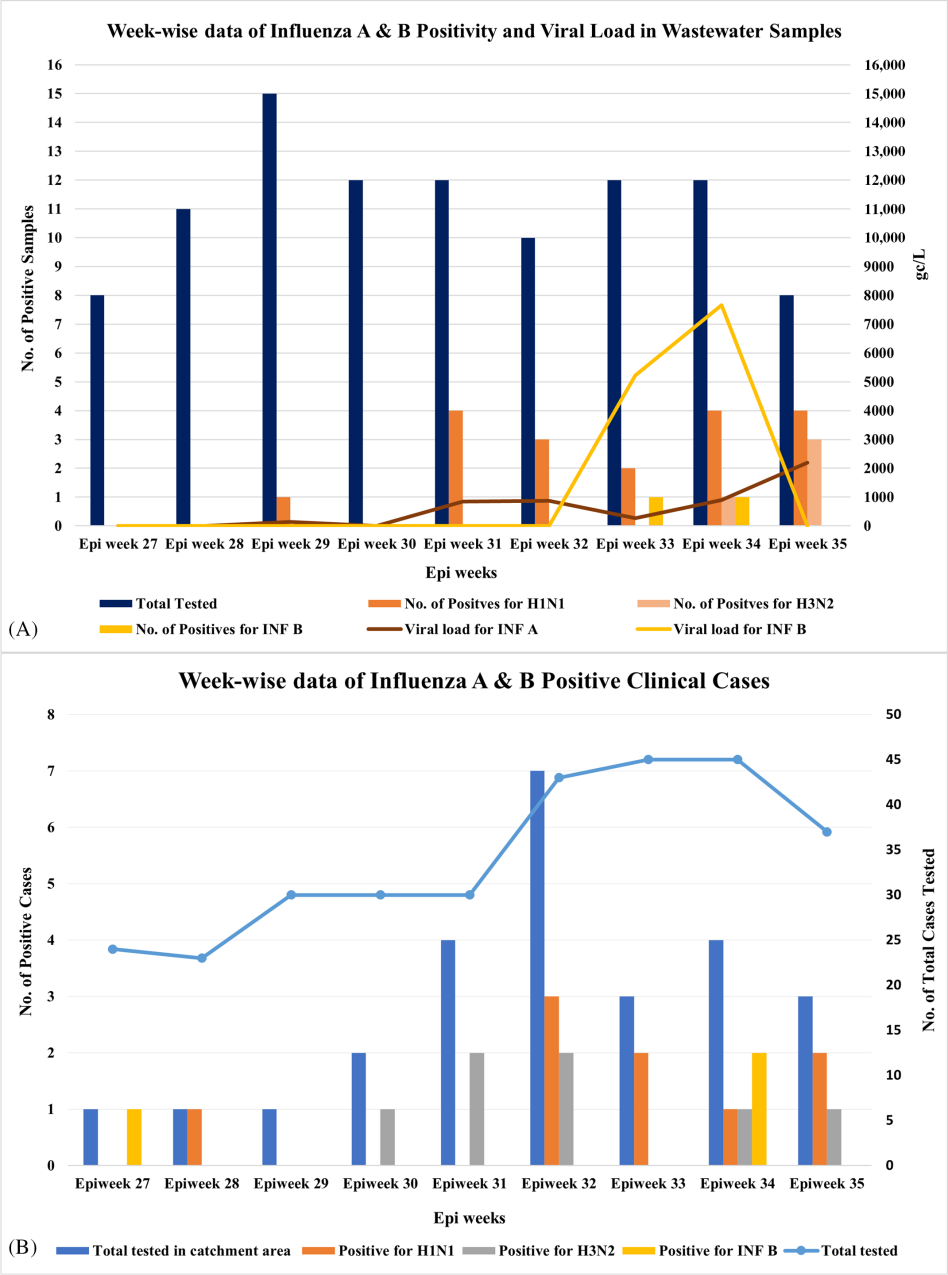
Week‐wise data of Influenza A Positivity in Wastewater and Clinical Sample. Week‐wise data for influenza A and influenza B in wastewater samples (A) and clinical cases (B) represented as a bar graph. The X‐axis indicates the epidemiological weeks, the left Y‐axis represents the number of positive samples, and the right Y‐axis indicates the viral load (A) in the wastewater treatment plant (WWTP) in gc/L. and total tested (B) clinical cases.

For analysis of weekly influenza A and B positivity, data during July and August epidemiological weeks (epi weeks) were used to allow for ease of comparison. July to August falls within the epi weeks 27 to 35. In July, only one WWTP sample tested positive for influenza A virus in epi week 29. However, in August (starting from epi week 31), there was a notable shift to an upward trend, as the number of positive samples increased, with the highest positivity occurring in epi weeks 34 and 35 with four positive samples. During epi weeks 34 and 35, eight influenza A(H1N1) positive samples were detected and four of influenza A(H1N1) positive samples tested positive for influenza A(H3N2) virus (one from epi week 34 and three from epi week 35). This pattern suggests a significant rise in Influenza A virus in the WWTPs during the month of August (Figure 2A).

Weekly analysis of Influenza B positive WWTPs samples revealed that from epi weeks 27 to 32, no samples tested positive for Influenza B, indicating a low prevalence of Influenza B virus in wastewater during that period. However, in August, during epi weeks 33 and 34, one sample tested positive in each week (Figure 2A).

### Viral load of influenza A and B virus in WWTP samples

During epi weeks 27 to 31, influenza A virus was not detected in wastewater, except epi week 29 were a viral load of around 1.4 x 10^2^ gc/L was detected. However, in epi week 31, the viral load increased to 8.4 x 10^2^ gc/L. Subsequently, in epi week 33, the viral load decreased slightly and increased again in epi week 34 to 9.0 x 10^2^ gc/L, and further increased to 2.2 x 10^3^ gc/L in the following epi week (Figure 2A). The highest and lowest viral load for influenza A was 2.2 x 10^3^ gc/L and 1.4 x 10^2^ gc/L, respectively.

During epi weeks 27 to 32, influenza B viral load remained consistently zero, indicating extremely low levels of Influenza B in the wastewater. However, in epi week 33, there was one positive sample with viral load of 5.2 x 10^3^ gc/L. The viral load increased to 7.7 x 10^3^ gc/L in epi week 34. Notably, the viral load dropped to zero in epi week 35 (Figure 2A).

### Week‐wise correlation of influenza A and B positivity in WWTP and clinical cases

The week‐wise Influenza A positivity in wastewater was correlated with the Influenza A cases from the catchment area (Figure 2B). Of the 26 influenza suspected clinical samples, 16 (61.5%) clinical samples tested positive for Influenza A virus. Among these positive samples, nine (56.3%) clinical samples were positive for influenza A(H1N1) virus, and seven (43.7%) clinical samples were positive for influenza A(H3N2) virus. The trends in clinical Influenza A cases and WWTPs samples was similar with a lower number of clinical cases and a corresponding low virus prevalence of virus in WWTP until August. However, in August (starting from epi week 31), there was an increase in positivity in both clinical cases **(**Figure 2B) and WWTP samples (Figure 2A), suggesting a rise of Influenza A virus in the community.

For correlation analysis, influenza A virus positivity in wastewater samples was compared with the number of influenza A cases in the same epi week, the week before, and the week after using Spearman's correlation test. The rate of positive samples was significantly correlated with the reported number of influenza A cases. A stronger correlation was found with the reported number of cases of the same epi week (*r* = 0.74), and the week before (*r* = 0.69). Influenza A(H1N1) cases and WWTP positivity showed strong correlation the week before (*r* = 0.80) and moderate correlation during the week (*r* = 0.44), whereas for influenza A(H3N2) virus, strong correlation was observed during the week (*r* = 0.6) and the week after (*r* = 0.67).

The week‐wise presence of Influenza B in wastewater samples exhibited a correlation with the number of clinical Influenza B cases. Of the 26 influenza suspected clinical cases from the catchment area, 3 (11.5%) clinical samples tested positive for Influenza B virus. During the epi weeks 27 to 32, only one case of Influenza B was reported, and no wastewater samples tested positive for Influenza B during this period. However, in epi week 34, two clinical cases of Influenza B were reported, and during the same period (epi weeks 33 and 34) Influenza B was also detected in wastewater samples (Figure 2).

### Mapping of influenza positivity in WWTP samples and clinical cases

**FIGURE 3:**
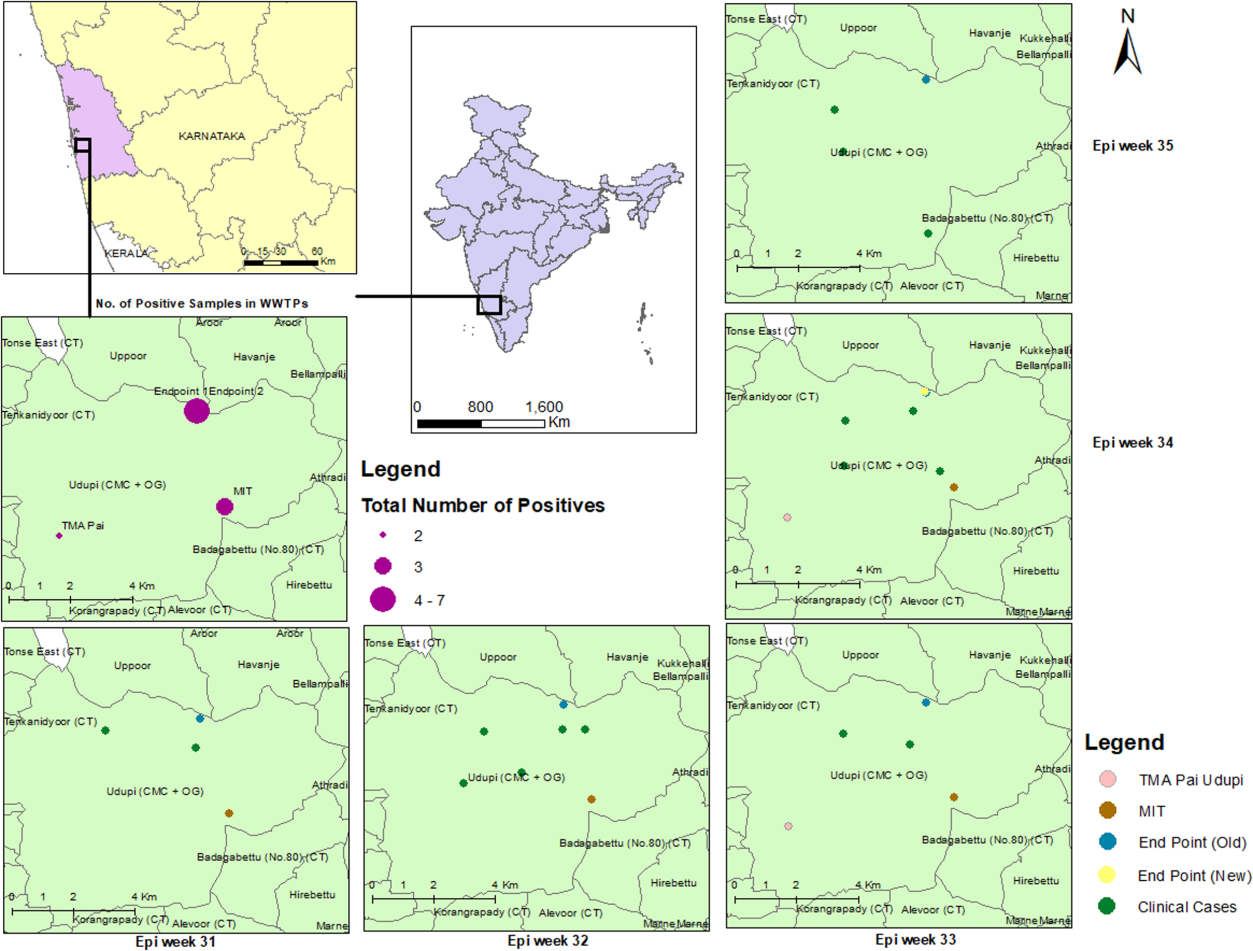
Week‐wise mapping of wastewater treatment plant (WWTPs) positive samples with clinical cases. Influenza A and Influenza B positive samples and cases have been mapped together. The size of the circle (purple) varies based on the total no. of positive samples. Wastewater positive samples have been colour coded to distinguish between different WWTPs: Pink for TMA Pai Udupi WWTP, brown for MIT WWTP, blue for End point (Old) WWTP, yellow for End point (New) WWTP, and green for clinical cases.

Throughout epi weeks 31–35, both WWTP samples and clinical cases tested positive for influenza virus. WWTPs and clinical cases from the catchment area were mapped to understand the spatial distance of WWTP and clinical cases. WWTP Endpoint new and Endpoint old are located within 1.5 km distance and catchment area includes both community and a tertiary hospital. Among the 18 influenza WWTP positive samples, eight were collected from Endpoint new and seven from Endpoint old. All the clinical cases were detected within 0.6–3.0 km distance from the WWTPs (Figure 3).

## DISCUSSION

The present study demonstrated a positivity rate of 18% for influenza A virus and 2% for influenza B virus in wastewater samples. All the 18 influenza A positive samples tested positive for influenza A (H1N1, 100%), although 22.22% of the influenza A samples were positive for both influenza A (H1N1) and A (H3N2) viruses. These findings are consistent with other studies, although the rates show considerable variations. For instance, a study in Ottawa, Canada, reported 60% positivity rate of for Influenza A virus in wastewater samples during February and March 2022 (Mercier et al., 2022). Similar research in Utrecht, the Netherlands, from October to February 2010, also reported positivity of 53.8% for Flu‐A and a 35.5% for panFlu‐A in sewage influent samples (Heijnen & Medema, 2011). In southeastern Germany from January 2022 to May 2022, positivity rates for Influenza B in wastewater samples ranged from 36 to 57.7% (Dumke et al., 2022). Some studies have consistently indicated the absence of Influenza B in wastewater samples during seasons of low viral activity (Mercier et al., 2022; Wolken et al., 2023). These variations highlight the influence of geographical and temporal factors on viral prevalence in wastewater, particularly in regions with temperate climates during the influenza season.

The current study was conducted in the Udupi district of Karnataka, India. India falls in the tropical region in Northern hemisphere. In India, influenza activity is reported throughout the year with some cities experiencing peaks in winter, whereas some cities experience peaks in July–September (Dhar et al., 2020). This study was conducted in Udupi district, where influenza activity is reported throughout the year with peaks in July (Jayaram et al., 2022) with the onset of monsoon, thus WWTP samples were collected in the month of July and August 2022.

Different methodologies have been used for viral enrichment and concentration from wastewater samples such as ultracentrifugation, precipitation, ultrafiltration and membrane adsorption (Zheng et al., 2022), however, most of them lack the scalability or use expensive instruments such as an ultracentrifuge (Zheng et al., 2023). In the current study WWTP samples were heat inactivated for 1 hour at 60°C. A previous study on SARS‐CoV‐2 has shown that heat inactivation prior to nucleic acid extraction may provide worker safety by inactivating the virus with no compromise of test result (Wu et al., 2020). Following pasteurization WWTP samples were centrifuged to remove solids and filtered using Whatman paper and PES membrane before proceeding for PEG precipitation. Wu et al., tested unfiltered, filtrate and solid material for presence of viral RNA and concluded that RNA levels in PEG precipitated pellet from filtrate was higher than unfiltered and solid fraction (Wu et al., 2020). PEG precipitation method for virus concentration is also widely used due to its cost‐effectiveness and scalability (Heijnen & Medema, 2011; Mercier et al., 2022).

Viral load of influenza A and B virus in wastewater was 1.4 x 10^2^–2.2 x 10^3^ gc/L and 5.2 x 10^3^–7.7 x 10^3^ gc/L, respectively. For viral load quantification of influenza A and B virus, purified PCR products were used since these products are easy to prepare and cost‐effective. Purified PCR products are most commonly used as standard for qPCR and study by Dhanasekaran et al. showed that PCR products stored at −20°C can be used within one week and may not lead to substantial variation in standard curve (Dhanasekaran et al., 2010). In this study, since PCR product was used as standard and influenza is an RNA virus the efficiency of reverse transcription could not be estimated and therefore, the viral load estimates of influenza A and B virus may not be absolute quantification. However, newer methodologies such as digital PCR (dPCR) also may not estimate RNA accurately since it detects absolute count of amplified cDNA targets (Sanders et al., 2013).

When comparing our results with previous studies, the result was generally consistent with the other reported ranges. For instances, study conducted in the Netherlands from October 2009 to February 2010 reported a viral load for Flu‐A at 2.6 x 10^5^ gc/L in sewage influent samples (Heijnen & Medema, 2011). In southeastern Germany, from January 2022 to May 2022, viral loads for Influenza A ranged, from t 9.8 x 10^2^ gc/L to the 3.0 x 10^5^ gc/L, indicating fluctuations during the sampling period (Dumke et al., 2022). For Influenza B, previous studies reported a wide range from 7.2 x 10^2^ gc/L to 2.1 x 10^7^ gc/L (Dumke et al., 2022). The observed differences in viral loads could be attributed to various factors including geographical location, sampling time, variations in wastewater sources and virus activity. Despite the inherent variability in viral load measurements across different regions and studies, our findings are consistent with the general trends observed globally.

WWTP sample positivity during July and August months showed correlation with the clinical cases reported from MIV in Udupi district. These findings align with other studies that observed a significant correlation between clinical cases and wastewater data (Heijnen & Medema, 2011; Jayaram et al., 2022; Mercier et al., 2022). During the month of August, there was an increase in the number of clinical cases positivity for Influenza virus at MIV. Simultaneously, there was a concurrent rise in the presence of Influenza virus in wastewater samples during the same month. In previous studies, the concentration of Influenza A virus in wastewater was closely related with the reported incidence rates of Influenza A cases (Wolfe et al., 2022). Our study has also demonstrated similar insight by showing a concurrent increase in influenza prevalence in both clinical and wastewater samples within the same month, coinciding with a rise in reported cases. A study by Mercier et al., showed that wastewater‐based surveillance can be used to predict the flu outbreak 17 days in advance before the traditional clinical surveillance (Mercier et al., 2022). However, since this study was conducted for only 2 months and the total number of positives were only 18 WWTP samples. Future studies on larger number of samples collected across various years would provide more sights on correlation of WWTP positivity with clinical cases. Furthermore, to accurately detect the beginning, peak, and end of a given influenza outbreak, year‐round surveillance of wastewater would be essential.

Our study provides preliminary data on the utility of WBE as a complementary surveillance tool for monitoring influenza A and influenza B viruses in India. This approach enhances the traditional clinical surveillance by offering real‐time insights into viral load dynamics within WWTPs, thus reflecting the community‐level infection trends.

## CONCLUSION

This study confirmed the presence of Influenza A and Influenza B viruses in wastewater samples collected from four WWTP. This finding has demonstrated that viruses are excreted by infected individuals and enter the wastewater system. The study implies the effectiveness of WBE as a surveillance tool for monitoring the presence of influenza viruses in a community. WBE provides concurrent detection viruses in the community along with reported clinical cases. The ability to monitor respiratory viruses in wastewater can aid in the early detection of changes in viral strains and guide vaccine development efforts.

One of the limitations of this study was the analysis of wastewater samples for only a two‐month period. Therefore, expanding the study to cover a longer period could provide a broader understanding of influenza virus dynamics in wastewater. The establishment of a long‐term WBE program could help monitor influenza viruses in wastewater continuously, identifying trends and seasonal variations over multiple years.

## AUTHOR CONTRIBUTIONS

**Sneka Panneerselvam:** Writing – original draft; formal analysis; investigation; methodology. **Athira Manayan Parambil:** Formal analysis. **Anup Jayaram:** Formal analysis. **Prasad Varamballi:** Formal analysis. **Chiranjay Mukhopadhyay:** Resources; supervision. **Anitha Jagadesh:** Conceptualization; investigation; writing – review and editing; validation; supervision; data curation.

## CONFLICT OF INTEREST STATEMENT

The authors affirm no conflict of interest.

## ETHICS STATEMENT

The Institutional Ethics Committee (IEC) reviewed and approved the study at Kasturba Medical College and Kasturba Hospital (Approved ID: IEC2: 672/2022).

## Data Availability

The data that support the findings of this study are available from the corresponding author upon reasonable request.
